# Non-controlling large shareholders and dynamic capital structure adjustment in China

**DOI:** 10.1371/journal.pone.0307066

**Published:** 2024-07-31

**Authors:** Jia Liao, Yun Zhan, Yu Yuan, Ailing Xu

**Affiliations:** 1 Business School, Huaqiao University, Quanzhou, Fujian, China; 2 College of Economics and Management FAFU, Fujian Agriculture and Forestry University, Fuzhou, Fujian, China; 3 School of Management, Jinan University, Guangzhou, Guangdong, China; Yunnan Technology and Business University, CHINA

## Abstract

Using the sample of Chinese A-share listed firms from 2010 to 2020, this study examines the impact of non-controlling large shareholders (NCLSs) on corporate capital structure adjustment. The results show that NCLSs significantly increase the dynamic capital structure adjustment speed and reduce capital structure deviation. NCLSs have an asymmetric influence on capital structure adjustment speed for different deviation directions, i.e. compared to the speed of upward adjustment after a downward deviation of the capital structure, the effect of NCLSs on the speed of downward adjustment of the capital structure after an upward deviation is stronger. Whether in state-owned enterprises (SOEs) or non-state-owned enterprises (NSOEs), NCLSs significantly increase the dynamic capital structure adjustment speed. However, compared with SOEs, NCLSs in NSOEs have a more significant positive impact on the dynamic capital structure adjustment speed. The mechanism analysis suggests that reducing agency costs and mitigating financing constraints serve as the important channels through which NCLSs influence the dynamic adjustment of capital structure. This paper not only enriches and improves the theoretical basis of dynamic capital structure adjustment, but also helps to deepen the understanding of dynamic capital structure adjustment of Chinese listed firms.

## 1. Introduction

Excessive leverage may transform the corporate financing model from hedge financing to speculative financing or even Ponzi financing, accumulating instability in the financial system and leading to systemic financial risks and financial crises [1]. To curb the further rise in leverage risk, the 2015 Central Economic Work Conference explicitly listed deleveraging as one of the five tasks of China’s supply-side structural reform. However, China suffers from both excessive debt and shortfalls in its economic development. The Chinese State Council urges the effective implementation of private investment policies, emphasizing that the difficulty of financing and expensive financing is still one of the prominent problems strongly reflected by private enterprises, and private enterprises have lots of intermediate procedures, high fees, and difficulties in applying for loans, and banks cherish loans, press loans, withdraw loans and cut off loans from time to time. In addition, banks dominate the Chinese financial system and bank loans have become the main source of external financing for firms [2,3]. However, due to government intervention, a large proportion of bank loans, especially long-term loans, are used by zombie firms [4], making it more difficult for other firms to obtain external financing through banks [5]. It can be seen that the slow speed of dynamic adjustment of capital structure is the main problem of China’s economic development, and the capital structure adjustment problems that exist in companies with different property rights are diametrically opposed.

Since Modigliani and Miller’s irrelevance of capital structure in 1958 [6], numerous studies have relaxed the strict assumptions of their theory from different perspectives and proposed many important theories, including the trade-off theory [7–11], the pecking order theory [12], the agency theory [13], and the dynamic adjustment of capital structure theory [14]. In present studies, the dynamic adjustment of capital structure theory has attracted much attention, which holds that there is a target capital structure, and when the actual capital structure deviates from this optimal capital structure, the firm tends to adjust to this goal [14–16]. Due to the reality of market frictions, firms have adjustment costs in the process of capital structure optimization [14], and whether and to what extent firms adjust their capital structure is subject to a trade-off between adjustment costs and benefits [15]. Flannery and Rangan [16] find that the presence of adjustment costs prevents firms from quickly adjusting their capital structure to the optimal one, but instead tend to make partial adjustments to the target capital structure at a certain rate each year.

Studies have shown that adjustment costs mainly arise from transaction costs caused by information asymmetry and agency problems and that internal agency costs and external financing costs are considered to constitute the main components of adjustment costs [17]. Morellec et al. [18] find that, compared to financing costs, agency costs have a significantly stronger effect in slowing down the rate of dynamic adjustment of a corporate capital structure. Lambrecht and Myers [19] argue that corporate capital structure adjustment is influenced by agency problems and that to maximize rents, management makes capital structure decisions that deviate from a predetermined optimal capital structure and choose to be overly indebted. Pindado and De La Torre [20] argue that corporate capital structure adjustment is mainly influenced by the agency problem of the controlling shareholder, who will adjust the corporate capital structure to deviate from the direction of maximizing the firm value for appropriation. On the one hand, an increase in corporate debt allows controlling shareholders to obtain more capital without diluting control [21], and it’s easier for them to engage in interest appropriation [19,22], resulting in corporate capital structure deviating from the optimal level. On the other hand, debt as a governance instrument can effectively restrain the tunneling behavior of controlling shareholders, and thus self-interested controlling shareholders tend to avoid the use of debt [23,24], leading to a capital structure that deviates from the optimal value. In China’s special institutional context, debt financing is an important way for firms to obtain external funds, and the protection mechanism for creditors’ interests is not yet perfect, so obtaining debt financing in the name of the firm and then transferring the funds becomes an important channel for controlling shareholders to seek personal gains [25]. Liu and Tian [22] find that firms with excess control rights have more excess leverage and their controlling shareholders use the resources for tunneling.

**Fig 1:**
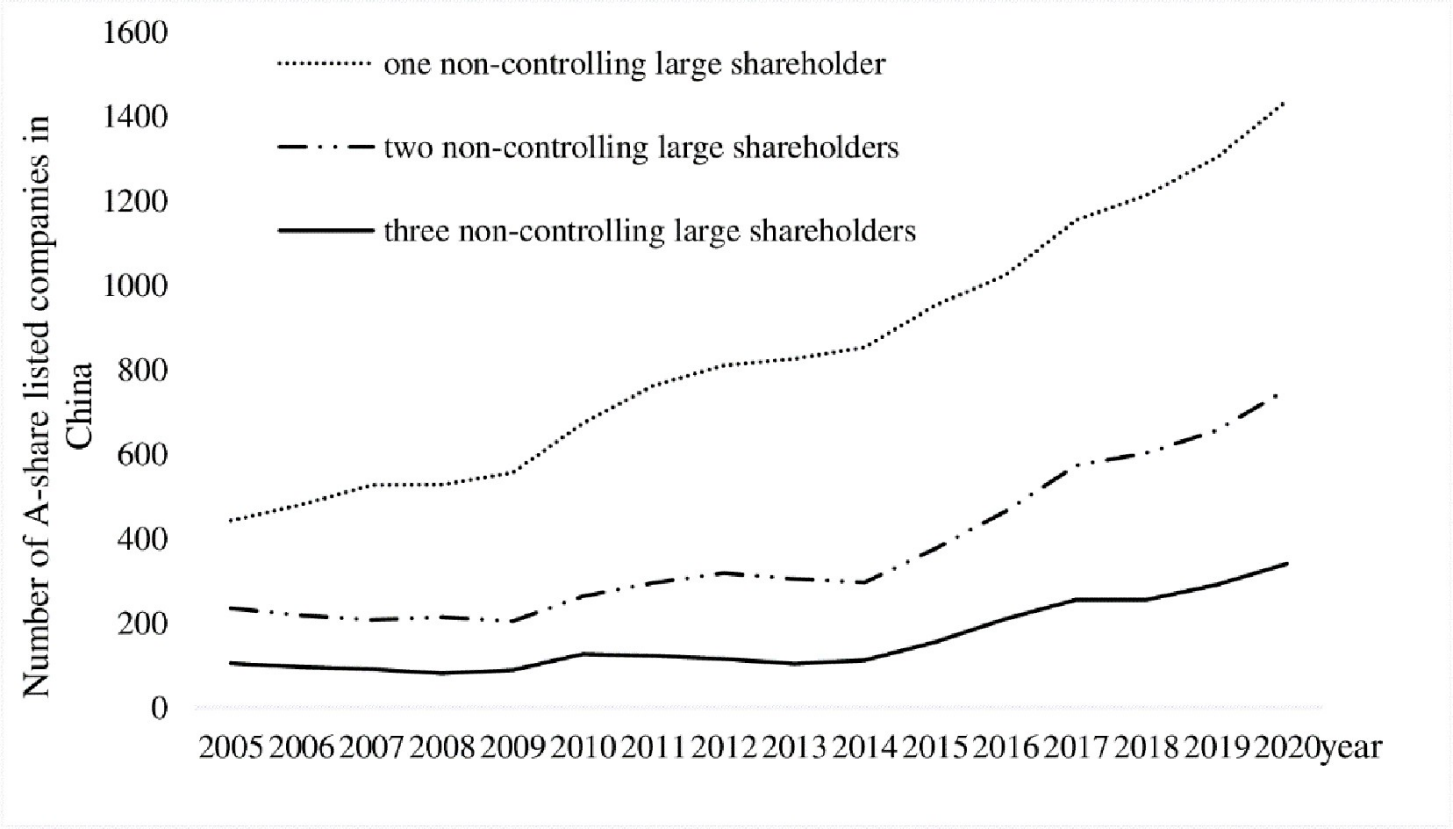
The number of listed firms with NCLSs in China from 2005 to 2020.

How to monitor managers’ opportunistic behavior while simultaneously restraining controlling shareholders’ self-interest tunneling is a key priority in the design of corporate governance mechanisms, as well as a key path to optimal corporate capital structure adjustment. In this study, we focus on the governance role of NCLSs. Specifically, we examine whether and how NCLSs affect corporate capital structure adjustment. NCLSs are a very special, common, and important presence under the current corporate governance model [26–28]. With the successful completion of China’s shareholding reform, removal of the restriction on non-floating shares, and in-depth promotion of mixed ownership reform, the ownership structure tends to be balanced and diversified and the number of listed firms with NCLSs shows a trend of increasing year by year (see Fig 1 The number of listed firms with NCLSs in China from 2005 to 2020). As the core point of equity checks and balances, NCLSs are an important counterweight to controlling shareholders, and their governance role in Chinese listed firms is gradually gaining attention. Studies show that NCLSs can participate in corporate governance not only by actively participating in decision-making [28] and sending directors to supervise [29,30] but also by "voting with feet" [27] or even just "threat with mouth" [31] to exert certain governance effects. Therefore, this paper takes a sample of A-share listed firms in China from 2010 to 2020, focuses the research perspective on NCLSs, and explores the optimization path of corporate capital structure adjustment from this important governance mechanism.

The research contributions and innovations of this paper are mainly reflected in the following aspects: Firstly, this paper refines the definition and measurement of NCLSs. Existing research on NCLSs mainly focuses on the perspective of multiple large shareholders’ equity structure, which mainly focuses on the "monitoring effect" and "collusion effect" of multiple large shareholders in theoretical analyses, and fails to differentiate NCLSs from the controlling shareholders and internal shareholders (managers and family members) in empirical tests, leading to inconsistent conclusions [32–36]. This paper fully considers the "identity" of large shareholders and treats concert parties with relationships as the same shareholder based on hand-collected information on shareholding relationships. Furthermore, this paper portrays the characteristics of NCLSs by three indicators: the existence of NCLSs, the number of NCLSs, and the sum of the shareholding ratios of NCLSs, to more accurately reflect the governance role played by NCLSs.

Secondly, this paper deepens the academic theories related to the governance of large shareholders. Previous studies on the governance effects of NCLSs have mainly focused on the theoretical analysis from two perspectives: "voting with hands" and "voting with feet". However, they have overlooked the significant role of "oral threat" as an important mechanism [27,28]. This paper addresses this gap by incorporating corporate governance by NCLSs through the voice mechanism (voting with hands), the exit threat mechanism (threat with mouth), and the exit mechanism (voting with feet) into the theoretical analysis framework, which provides a clearer and more complete explanation for how NCLSs exert their corporate governance effects.

Thirdly, this paper enriches and extends the research on the influence of NCLSs on corporate financing decisions. Based on multiple large shareholders’ equity structure perspectives, existing studies have examined how NCLSs affect the firm’s capital structure [33], debt maturity structure [32], financing costs [37], and financing constraints [34]. The above studies mainly focus on the static perspective, while this paper extends the research perspective to dynamic capital structure, which serves as a valuable complement and extension to the existing research on the impact of NCLSs on firms’ financing decisions.

Fourthly, this paper enriches the literature on corporate capital structure adjustment. Existing studies mainly examine the causes and mitigating measures of corporate capital structure deviation from the perspectives of macroeconomic or institutional environments such as Supply-Side Structural Reform [38], tax enforcement [39], generalized trust [40], social network [41,42], bank competition [43] and business environment [44]. From the perspective of corporate governance, this paper examines the impact of NCLSs, an important governance mechanism, on the adjustment of enterprise capital structure and the mechanism of its role, which contributes to a deeper understanding of the dynamic adjustment of the capital structure of Chinese firms and provides new ideas for effectively promoting the realization of the goal of "deleveraging" of firms.

## 2. Literature review and research hypotheses

### 2.1 NCLSs and capital structure adjustment

Corporate capital structure choice is an important decision concerning solvency, profitability, and corporate value [16]. Existing studies mainly focus on capital financing decisions [45–47], research on dynamic capital structure adjustment still remains limited. This paper mainly investigates capital structure adjustment from the role of NCLSs.

NCLSs have incentives to accelerate the dynamic capital structure adjustment speed and reduce the extent to which the firm’s actual capital structure deviates from the target capital structure. Unlike small and medium-sized shareholders or retail investors who focus on the investment returns from short-term stock price increases and decreases, NCLSs pay more attention to the long-term high-quality development of the firm to bring about a continuous and stable increase in stock price [28]. The target capital structure reflects the optimal match between risk and return and is also a form of expression of corporate value maximization [16]. Therefore, NCLSs will be willing to spend a lot of time and effort paying attention to whether the corporate capital structure decisions are consistent with the expectations associated with dynamic trade-off theory. In addition, NCLSs hold a higher share of equity in listed firms, and their interests are particularly harmed by the self-interest of controlling shareholders and managers in distorting capital structure decisions for value appropriation. In order to avoid wealth loss, they have a strong incentive to supervise and restrain the capital structure decisions of the firm, thus speeding up the corporate capital structure adjustment to the target level and reducing the actual capital structure deviation from the target capital structure.

In addition to being motivated, NCLSs have sufficient ability to accelerate the dynamic capital structure adjustment speed and reduce the extent to which the actual capital structure of the firm deviates from the target capital structure. NCLSs can play an effective governance role in mitigating the problem of capital structure deviation and slow adjustment caused by agency costs. NCLSs with higher shareholdings have the opportunity to appoint directors or executives to the firm to participate directly in strategic management and operational decisions, or to request the convening of an extraordinary shareholders’ meeting following the Firm Law to stop the self-interested behavior of controlling shareholders and management in distorting capital structure decisions for value appropriation. Studies have shown that NCLSs’ participation and exercise of rights can effectively stop controlling shareholders’ and management’s self-interest behavior [48]. Directors assigned by NCLSs also act as a good check on controlling shareholders and managers [27], and their active voice in board motions can help reduce agency costs and improve operational efficiency and corporate value [49]. Even when the direct monitoring role of "voting with hands" fails, NCLSs can still bargain with controlling shareholders and managers to restrain their self-interested behavior by releasing a credible "threat of exit". Numerous studies have shown that NCLSs’ exit threat can effectively curb management agency costs or control shareholders’ agency costs [50]. Therefore, under the supervision and constraint of NCLSs, the self-interest motives of controlling shareholders and managers will be moderated, and they will take into account the interests of listed firms and non-controlling shareholders when making capital structure decisions, and will not intentionally distort the firm’s debt level for the purpose of resource transfer or avoiding creditor supervision, so that the corporate capital structure will converge to the target capital structure in time.

Besides, the governance role of NCLSs can also reduce the concerns of creditors and equity investors about the risk of the firm, and promote rapid access to debt financing or equity financing at a reasonable cost, thus mitigating the problem of capital structure deviation and slow adjustment caused by financing constraints. Numerous studies have confirmed that the presence of NCLSs in a firm can optimize the debt maturity structure [32], reduce financing costs [37], and effectively alleviate the financing constraints faced by the firm [34]. Therefore, under the influence of NCLSs, the improvement of the firm’s financing capacity and the reduction of financing costs can help accelerate the dynamic adjustment of the corporate capital structure and reduce the extent to which the firm’s actual capital structure deviates from the target capital structure. In addition, NCLSs help to build institutionalized communication channels and participation platforms [51], these help NCLSs jointly participate in the formulation and implementation of corporate capital structure decisions. As a result, insiders have a higher level of knowledge about the capital structure, which in turn allows them to identify the target capital structure and the degree of actual capital structure deviation, and to quickly adjust this to make it converge to the target capital structure. Based on the above analysis, this paper proposes the following two hypotheses.

*H1*: *NCLSs increase the dynamic capital structure adjustment speed of the firm*.
*H2*: *NCLSs reduce the extent to which the actual capital structure deviates from the target capital structure of the firm*.

### 2.2 NCLSs and asymmetric capital structure adjustment of the firm

According to trade-off theory, the optimal capital structure of a firm should be the optimal point between the tax saving benefits and the cost of financial distress from debt financing [7–11]. Due to the presence of market friction, the actual capital structure of the firm inevitably deviates from the target level. When the actual capital structure is higher than the target level (upward deviation), the firm has a higher probability of falling into a financial crisis or even bankruptcy, and the marginal financial distress cost of increasing debt is much higher than the marginal tax saving benefit. Conversely, when the actual capital structure is lower than the target level (downward deviation), the marginal tax saving benefit obtained by increasing debt is higher than the marginal financial distress cost. Behavioral finance theory suggests that decision makers’ perceptions of the utility of equal costs and benefits are usually asymmetric, and they are more "loss averse" in the face of equal cost increases and benefit decreases [52]. Specifically, concerning the corporate capital structure decision, a downward deviation of the actual capital structure from the target level will lead to a reduction in tax saving benefits, while an upward deviation will lead to an increase in financial distress costs. Under the loss aversion condition, even if the actual capital structure deviates to the same extent up or down, the utility loss caused by deviations in different directions will differ significantly, and thus the incentive and urgency to make convergence adjustments will be different.

NCLSs are similarly more sensitive to potential losses than to reductions in equivalent returns, so this paper argues that NCLSs have an asymmetric influence on capital structure adjustment speed for different deviation directions. NCLSs hold a higher share of equity in listed firms, and their wealth is closely related to the financial distress cost and tax-saving benefits of the firm’s liabilities, thus they will consciously pay attention to the direction of deviation of the corporate capital structure from the target. NCLSs can detect and identify the target capital structure of the firm and determine the deviation of the actual capital structure from the target level in time, and when facing the capital structure deviation in different directions, NCLSs with asymmetric utility perception will have different governance effects. Specifically, when the actual capital structure of the firm is higher than the target level, the loss aversion motive induces NCLSs to be extremely averse to the risk of financial crisis or even bankruptcy caused by over-indebtedness. NCLSs have a strong incentive and sufficient ability to monitor and discipline the over-indebtedness of the controlling shareholder and managers for selfish appropriation motives, and to prompt the firm to adjust its capital structure downward to the target capital structure. It has been shown that the equity checks and balances of NCLSs in firms with multiple large shareholders can significantly inhibit over-indebtedness [33] and thus help accelerate the downward adjustment of the capital structure. Conversely, when the actual capital structure of the firm is lower than the target level, increasing the level of indebtedness can bring tax savings but increase the risk of bankruptcy and the cost of financial distress. As a result, the governance effect on the dynamic capital structure adjustment speed is significantly weaker. Based on the above analysis, we propose the following hypothesis.

*H3*: *Compared to the speed of upward adjustment after a downward deviation of the capital structure*, *the effect of NCLSs on the speed of downward adjustment of the capital structure after an upward deviation is stronger*.

### 2.3 The moderating effect of ownership structure on the relationship between NCLSs and the dynamic capital structure adjustment speed of the firm

Ownership structure has been an important topic that cannot be ignored in studying the financing behavior of China’s listed firms [53–55]. This paper argues that the governance role of NCLSs on the dynamic capital structure adjustment should be different depending on the ownership structure of the firm, and the NCLSs of non-state-owned enterprises (NSOEs) have a more significant role in enhancing dynamic capital structure adjustment speed compared with state-owned enterprises (SOEs) for the following reasons. First, in addition to the financial goal of preserving and increasing the value of state-owned assets, SOEs also carry more non-financial goals of maximizing social interests [56]. In contrast, NSOEs are more likely to fall into financial crises, and the loss aversion motive makes NCLSs more motivated to intervene in the dynamic capital structure adjustment speed of the firm. Second, the "close relationship" between SOEs and the government creates a superior internal and external environment and conditions for their financing. The government tends to provide credit resources to SOEs, and the "underwriting" role of national or local governments also makes banks and other financial institutions more willing to grant loans to SOEs [54]. In contrast, NSOEs suffer from more severe "discrimination" when it comes to credit financing [53], and the risk of bankruptcy and liquidation is higher in case of a financial crisis, so NCLSs have more incentives to promptly adjust the capital structure of NSOEs to the target level. Third, the unique "political color" of SOEs makes their behavioral decisions inevitably influenced by government intervention, and most SOEs are involved in important areas related to the country’s livelihood, so they are naturally closely watched by the public, and insiders of SOEs cannot directly encroach on the interests of outside investors. In contrast, NSOEs are less affected by government intervention, and the second type of agency conflict is more serious, and controlling shareholders are more likely to obtain private benefits through tunneling behavior [57], so NCLSs have more incentives to supervise and restrain, and thus significantly accelerate the dynamic capital structure adjustment. Fourth, operators of SOEs are mostly appointed or reassigned by their superiors [58] and pay more attention to the implementation of national policies in their business decisions, and also pay special attention to promotion incentives during their careers. Therefore, it is difficult for the NCLSs of SOEs to intervene in the appointment and removal of management by "voting with hands", and their attempts to restrain management behavior through the "threat of exit" are difficult to achieve. In contrast, NCLSs can effectively motivate NSOEs to adjust their capital structure to the optimal level in a timely manner, as most of the operators of NSOEs are selected and recruited through the market, and they care about the performance level and future share price trend of the enterprises. Based on the above analysis, we propose the following hypothesis.

*H4*: *Compared with SOEs*, *NCLSs in NSOEs have a more significant positive impact on the dynamic capital structure adjustment speed*.

## 3. Methodology

### 3.1 Sample selection and data sources

The data used in this paper come from the China Stock Market and the Accounting Research (CSMAR) database. Table 1 presents our data selection process. We initially select A-share firms listed on the Shanghai and Shenzhen Stock Exchanges in China during 2010–2020 as the research sample. To ensure the accuracy and stability of the data, we exclude the following firms: (a) firms in the financial industry, (b) ST, *ST firms, and (c) firms with leverage greater than 1 or negative owner’s equity, (d) firms with less than two consecutive years of observations, (e) controlling shareholder’s shareholdings is less than 5% after aggregating the shareholdings of concert parties, and (f) firms with missing values. The final sample includes 26,001 firm-year observations of 3,538 unique firms. All continuous variables are winsorized to minimize the effects of outliers.

**Table 1:** Sample selection.

Sample selection process	Observations
A-share firms listed on the Shanghai and Shenzhen Stock Exchanges in China during 2010–2020	32,561
Delete: Firms in the financial industry	(718)
Delete: ST, *ST firms	(1,487)
Delete: Firms with leverage greater than 1 or negative owner’s equity	(76)
Delete: Firms with less than two consecutive years of observations	(2,029)
Delete: Firms with CS’s shareholdings is less than 5% or CS is not the largest shareholder	(46)
Delete: Firms with missing values	(2,204)
Final sample	26,001

### 3.2 Measurement model and variable definitions

#### 3.2.1 Measures of target capital structure and capital structure adjustment speed

Drawing on the relevant literature [16,17,41,42,59], we measure the dynamic adjustment of capital structure based on a standard partial adjustment model, as shown in model (1).

$$Lev_{i,t}-Lev_{i,t‐1}=\alpha (Le{v^{*}}_{i,t}-Lev_{i,t‐1})+\varepsilon_{i,t}$$ (1)

where *Lev*_*i*,*t*_, *Lev*_*i*,*t-1*_ denote the actual capital structure of firm i in year t and year t-1, respectively, calculated as the ratio of the total liabilities to total assets. Lev^*^_i,*t*_ denotes the target capital structure of firm i in year t. The regression coefficient *α* indicates that the difference between the actual capital structure and the target capital structure of the firm narrows at an average rate of *α* per year, i.e., the dynamic capital structure adjustment speed.

Since Lev^*^_i,*t*_ is not directly available, we follow existing studies [16,17,59] and select a series of firm characteristic variables that may have an impact on the capital structure (see Table 2 for details) as well as controlling for firm, industry and year fixed effects, the target capital structure is fitted as shown in model (2).


$$Le{v^{*}}_{i,t}=\beta_{1}Size_{i,t‐1}+\beta_{2}Roa_{i,t‐1}+\beta_{3}Growth_{i,t‐1}+\beta_{4}Dep_{i,t‐1}+\beta_{5}Pota_{i,t‐1}+\beta_{6}Indlev_{i,t‐1}$$ (2)

**Table 2:** Variable definitions and calculation methods.

Variables	Calculation methods	Data source
*Lev*	The ratio of the total liabilities to total assets	China Stock Market Financial Database–Financial Indices in the CSMAR database
*ΔLev*	Corporate actual capital structure in year t minus actual capital structure in year t-1	
*Dev*	Corporate target capital structure in year t minus actual capital structure in year t-1	
*D* ^*above*^	Take the value of 1 if the actual capital structure of firm i in year t-1 is higher than the target capital structure in year t and 0 otherwise	
*Dum*	A dummy variable that takes the value of 1 if there is at least an NCLS in the firm, and 0 otherwise	
*Num*	The total number of NCLSs in the firm	China Listed Firm’s Shareholders Research Database in the CSMAR database
*Ratio*	The sum of the shareholding ratios of all NCLSs in the firm	
*NSoe*	A dummy variable that takes the value of 1 if the firm is a non-state-owned enterprise and 0 otherwise.	China Listed Firm’s Equity of Nature Research Database in the CSMAR database
*Size*	Natural logarithm of total assets	China Stock Market Financial Statements Database in the CSMAR database
*Roa*	The ratio of the net profit to total assets	China Stock Market Financial Database–Financial Indices in the CSMAR database
*Growth*	The growth rate of the firm’s operational revenue	
*Dep*	Depreciation of fixed assets/total assets	China Stock Market Financial Statements Database in the CSMAR database
*Pota*	The ratio of net fixed assets plus net inventories to total assets	
*Indlev*	Average capital structure of the industry	Chinese Listed Firms’ Industry Financial Indicators Research Database in the CSMAR database

Next, model (2) is substituted into model (1) to obtain model (3), as follows.


$$Lev_{i,t}=(1-\alpha )Lev_{i,t‐1}+\alpha \beta_{1}Size_{i,t‐1}+\alpha \beta_{2}Roa_{i,t‐1}+\alpha \beta_{3}Growth_{i,t‐1}+\alpha \beta_{4}Dep_{i,t‐1}+\alpha \beta_{5}Pota_{i,t‐1}+\alpha \beta_{6}Indlev_{i,t‐1}+\varepsilon_{i,t}$$ (3)

Then, we estimate the regression of model (3), from which we obtain (1−*α*) and the values of *αβ*_*1*_*~αβ*_*6*_, which in turn allow us to calculate the values of *β*_*1*_*~β*_*6*_. The values of Lev^*^_i,*t*_ can be calculated by substituting *β*_*1*_*~β*_*6*_ into model (2).

#### 3.2.2 Measurement model of H1

To verify H1, i.e. to examine the impact of NCLSs on the dynamic capital structure adjustment speed, we substitute the calculated value of the target capital structure Lev^*^_i,*t*_ into model (1), on which the *Ncls*_*i*,*t*_ is further incorporated, as shown in model (4) using panel fixed effects.

$$\Delta Lev_{i,t}=(\gamma_{0}+\gamma_{1}\times Ncls_{i,t})\times Dev_{i,t}+\varepsilon_{i,t}$$ (4)

where *ΔLev*_*i*,*t*_ *= Lev*_*i*,*t*_−*Lev*_*i*,*t-1*_, denotes the actual capital structure adjustment i in year t. *Dev*_*i*,*t*_ = (Lev^*^_i,*t*_ −*Lev*_*i*,*t-1*_), denotes the actual capital structure deviation of firm i in year t-1 from the target capital structure in year t. *Ncls*_*i*,*t*_ denotes the NCLSs of firm i in year t. We construct the following three variables to measure NCLSs: (a) *Dum*: takes the value of 1 if there is at least an NCLS in the firm, and 0 otherwise, (b) *Num*: represents the total number of NCLSs in the firm, and (c) *Ratio*: denotes the sum of the shareholding ratios of all NCLSs in the firm. The coefficient *γ*_1_ of *Ncls*_*i*,*t*_×*Dev*_*i*,*t*_ is the main parameter to be estimated, and if H1 holds, then its coefficient estimate should be significantly positive.

#### 3.2.3 Measurement model of H2

To verify H2, i.e. to examine the effect of NCLSs on the degree of capital structure deviation, the following panel fixed effects model is constructed.


$$Dev_{i,t}=\delta_{0}+\delta_{1}Ncls_{i,t}+\delta_{2}Size_{i,t‐1}+\delta_{3}Roa_{i,t‐1}+\delta_{4}Growth_{i,t‐1}+\delta_{5}Dep_{i,t‐1}+\delta_{6}Pota_{i,t‐1}+\delta_{7}Indlev_{i,t‐1}+\varepsilon_{i,t}$$ (5)

The coefficient *δ*_*1*_ of *Ncls*_*i*,*t*_ is the main parameter to be estimated, and if H2 holds, then its coefficient estimate should be significantly negative.

#### 3.2.4 Measurement model of H3

To verify H3, i.e. to examine the effect of NCLSs on the asymmetric capital structure adjustment speed, the following panel fixed effects model is constructed.

$$\Delta Lev_{i,t}=\zeta_{0}+\zeta_{1}Dev_{i,t}\times {D_{i,t}}^{above}+\zeta_{2}{D_{i,t}}^{above}+\zeta_{3}Ncls_{i,t}\times Dev_{i,t}\times {D_{i,t}}^{above}+\zeta_{4}Ncls_{i,t}\times {D_{i,t}}^{above}+\varepsilon_{i,t}$$ (6)

where *D*_*i*,*t*_^*above*^ is an upward deviation dummy variable that takes the value of 1 if the actual capital structure of firm i in year t-1 is higher than the target capital structure in year t and 0 otherwise. The coefficient *ζ*_*3*_ of *Ncls*_*i*,*t*_×*Dev*_*i*,*t*_×*D*_*i*,*t*_^*above*^ is the main parameter to be estimated, and if H3 holds, then its coefficient estimate should be significantly positive.

#### 3.2.5 Measurement model of H4

To verify H4, i.e. to examine the moderating effect of ownership structure on the relationship between the NCLSs and the dynamic capital structure adjustment speed, this paper further incorporates *NSoe*_*i*,*t*_ based on model (4), as shown in model (7) using panel fixed effects.

$$\Delta Lev_{i,t}=(\eta_{0}+\eta_{1}\times Ncls_{i,t}\times NSoe_{i,t})\times Dev_{i,t}+\varepsilon_{i,t}$$ (7)

where *NSoe*_*i*,*t*_ is a dummy variable that takes the value of 1 if the firm is a non-state-owned enterprise and 0 otherwise. The coefficient *η*_*1*_ of *Ncls*_*i*,*t*_×*NSoe*_*i*,*t*_×*Dev*_*i*,*t*_ is the main parameter to be estimated, and if H4 holds, then this coefficient estimate should be significantly positive.

## 4. Empirical analysis

### 4.1 Descriptive statistics

The descriptive statistics in Table 3 show that the mean and median of *Lev are* 0.441 and 0.437, respectively, with a standard deviation of 0.203 and maximum and minimum values of 0.869 and 0.053, indicating that the sample firms are, on average, at a medium level of indebtedness and that the level of capital structure varies significantly among firms. The mean and median of *ΔLev* are 0.010 and 0.008, the standard deviation is 0.082, and the maximum and minimum values are 0.802 and -0.801, respectively, indicating that the actual capital structure adjustment of the sample firms is relatively small on average, however, the differences in the directions and degrees of adjustment among different firms are obvious. The mean values of *Dum* and *Ratio* are 0.504 and 0.082 respectively, indicating that about 50.4% of the firms in the sample have NCLSs, but the shareholding is generally not high, revealing that "One Big Share Alone" is particularly prominent in China’s capital market. The maximum value of *Num* is 9, which indicates that the number of NCLSs in individual firms is high and the distribution of NCLSs varies significantly among firms.

**Table 3:** Descriptive statistics.

Variable	Observations	Mean	Median	Sd	Min	Max
*Lev*	26001	0.441	0.437	0.203	0.053	0.869
*ΔLev*	26001	0.010	0.008	0.082	-0.801	0.802
*Dev*	26001	0.010	0.011	0.155	-0.665	0.512
*Dum*	26001	0.504	1.000	0.500	0.000	1.000
*Num*	26001	0.736	1.000	0.902	0.000	9.000
*Ratio*	26001	0.082	0.050	0.106	0.000	0.808
*Size*	26001	22.140	21.960	1.275	19.500	25.790
*Roa*	26001	0.040	0.036	0.051	-0.160	0.193
*Growth*	26001	0.190	0.113	0.446	-0.567	2.905
*Dep*	26001	0.371	0.357	0.181	0.026	0.816
*Pota*	26001	0.020	0.016	0.015	0.000	0.071
*Indlev*	26001	0.417	0.396	0.104	0.229	0.693

### 4.2 Correlation analysis

The correlation analysis between the variables in Table 4 shows that the Pearson correlation coefficient between *ΔLev* and *Dev* is 0.232 and the Spearman correlation coefficient is 0.188, both of which are significant at the 1% level, initially indicating that the firm does dynamically adjust towards the target capital structure. Both coefficients of *Lev* and *Roa* are significantly negative at the 1% level, indicating that the risks associated with high financial leverage will negatively affect the profitability of the firm. Since this paper focuses on the coefficient estimates of *Ncls×Dev*, and *ΔLev* is also significantly correlated with the remaining control variables, the research hypothesis of this paper needs to be tested by multiple regression analysis.

**Table 4:** Correlation matrix.

	*Lev*	*ΔLev*	*Dev*	*Dum*	*Num*	*Ratio*	*Size*	*Roa*	*Growth*	*Dep*	*Pota*	*Indlev*
*Lev*		0.159***	-0.801***	-0.010*	-0.017***	0.001	0.488***	-0.396***	0.026***	0.294***	-0.034***	0.357***
*ΔLev*	0.184***		0.188***	0.001	-0.001	-0.002	-0.088***	0.009	0.019***	-0.080***	-0.052***	-0.052***
*Dev*	-0.786***	0.232***		0.013**	0.022***	0.019***	-0.188***	0.294***	-0.035***	-0.180***	-0.009	-0.195***
*Dum*	-0.012**	-0.003	0.013**		0.945***	0.924***	0.021***	0.017***	0.023***	-0.042***	0.006	-0.017***
*Num*	-0.025***	-0.013**	0.028***	0.810***		0.951***	0.025***	0.015**	0.029***	-0.043***	0.003	-0.014**
*Ratio*	0.008	-0.009	0.026***	0.766***	0.861***		0.049***	0.012*	0.017***	-0.022***	0.026***	0.006
*Size*	0.486***	-0.073***	-0.127***	0.039***	0.043***	0.109***		-0.074***	0.046***	0.155***	-0.014**	0.244***
*Roa*	-0.340***	0.026***	0.229***	0.005	-0.001	0.008	-0.025***		0.295***	-0.204***	-0.057***	-0.161***
*Growth*	0.050***	0.002	-0.036***	0.034***	0.049***	0.030***	0.046***	0.187***		-0.068***	-0.113***	-0.039***
*Dep*	0.304***	-0.087***	-0.160***	-0.048***	-0.045***	-0.009	0.177***	-0.154***	-0.057***		0.516***	0.174***
*Pota*	-0.011*	-0.054***	-0.027***	-0.004	-0.007	0.030***	0.027***	-0.066***	-0.102***	0.508***		-0.106***
*Indlev*	0.398***	-0.045***	-0.204***	-0.024***	-0.018***	0.011*	0.277***	-0.121***	0.018***	0.214***	-0.165***	

Note(s): Lower-triangular cells report Pearson’s correlation coefficients, upper-triangular cells are Spearman’s rank correlation

*** p<0.01

** p<0.05

* p<0.1.

### 4.3 Empirical results

#### 4.3.1 The effect of NCLSs on dynamic capital structure adjustment speed

Table 5 reports the overall trend in the dynamic capital structure adjustment speed of the firms during the sample period and the effect of NCLSs. As can be seen from column (1) of Table 5, the regression coefficient of *Dev* is 0.402 and is significant at the 1% level, indicating that the sample firms do exhibit a trend of adjustment toward the target capital structure and that the average adjustment speed is 40.2% which is consistent with the expectations associated with dynamic trade-off theory. In columns (2)-(4) of Table 5, the regression coefficients of *Dev* are always significantly positive at the 1% level, and the regression coefficients of *Dev*×*Dum*, *Dev*×*Num*, and *Dev*×*Ratio* are 0.076, 0.046, and 0.333, respectively, and all are significant at the 1% level, indicating that when there are NCLSs in the firm, the dynamic capital structure adjustment speed is significantly faster, and the higher the number of NCLSs and their shareholdings, the faster the dynamic capital structure adjustment speed. Therefore, H1 is verified.

**Table 5:** The effect of NCLSs on dynamic capital structure adjustment speed.

	(1)	(2)	(3)	(4)
	*ΔLev*			
*Dev*	0.402***	0.361***	0.363***	0.371***
	(70.213)	(49.379)	(53.237)	(54.090)
*Dev×Dum*		0.076***		
		(9.007)		
*Dev×Num*			0.046***	
			(10.215)	
*Dev×Ratio*				0.333***
				(7.929)
*_cons*	0.013	0.016	0.016	0.015
	(0.701)	(0.916)	(0.917)	(0.833)
Year	YES	YES	YES	YES
Firm	YES	YES	YES	YES
Industry	YES	YES	YES	YES
Observations	26001	26001	26001	26001
Adjusted *R*^*2*^	0.059	0.062	0.063	0.062

Note(s): *p < 0.1

**p < 0.05

***p < 0.01.

#### 4.3.2 The effect of NCLSs on capital structure deviation

Table 6 reports the regression results of the effect of NCLSs on capital structure deviation. The results show that the regression coefficients of *Dum* and *Num* are -0.004 and -0.002, respectively, and both are significant above the 1% level, and the regression coefficient of *Ratio* is -0.015 and significant at the 5% level, indicating that the degree of capital structure deviation is significantly reduced when there are NCLSs in the firm, and the higher the number of NCLSs and their shareholdings, the lower the capital structure deviation. Therefore, H2 is verified.

**Table 6:** The effect of NCLSs on capital structure deviation.

	(1)	(2)	(3)
	*Dev*		
*Dum*	-0.004***		
	(-2.721)		
*Num*		-0.002***	
		(-3.079)	
*Ratio*			-0.015**
			(-2.070)
*Size*	-0.009***	-0.009***	-0.009***
	(-7.682)	(-7.672)	(-7.694)
*Roa*	-0.175***	-0.175***	-0.175***
	(-15.105)	(-15.092)	(-15.101)
*Growth*	-0.003**	-0.003**	-0.003**
	(-2.517)	(-2.450)	(-2.488)
*Dep*	-0.065***	-0.065***	-0.065***
	(-11.934)	(-11.960)	(-11.917)
*Pota*	-0.107	-0.109	-0.110
	(-1.410)	(-1.430)	(-1.437)
*Indlev*	0.052***	0.052***	0.052***
	(2.864)	(2.865)	(2.874)
*_cons*	0.335***	0.335***	0.335***
	(11.172)	(11.151)	(11.153)
Year	YES	YES	YES
Firm	YES	YES	YES
Industry	YES	YES	YES
Observations	26001	26001	26001
Adjusted *R*^*2*^	0.027	0.027	0.027

Note(s): *p < 0.1

**p < 0.05

***p < 0.01.

#### 4.3.3 The effect of NCLSs on asymmetric capital structure adjustment speed

Table 7 reports the regression results on the effect of NCLSs on the asymmetric capital structure adjustment speed. The results in columns (1)-(3) of Table 7 using the upward deviation in capital structure subsample show that the regression coefficients of *Dev*×*Dum*, *Dev*×*Num*, and *Dev*×*Ratio* are 0.179, 0.128, and 1.053, respectively, and all are significant at the 1% level. The results in columns (4)-(6) of Table 7 using the downward deviation in the capital structure subsample show that the regression coefficients of *Dev*×*Dum*, *Dev*×*Num*, and *Dev*×*Ratio* are not significant. The results in columns (7)-(9) of Table 7 show that the regression coefficients of *Dum×Dev×D*^*above*^, *Num×Dev×D*^*above*^, and *Ratio×Dev×D*^*above*^ are 0.179, 0.105, and 0.953, respectively, and all are significant at the 1% level. The above results show that compared to the speed of upward adjustment after a downward deviation of the capital structure, the effect of NCLSs on the speed of downward adjustment of the capital structure after an upward deviation is stronger, i.e., H3 is verified.

**Table 7:** The effect of NCLSs on asymmetric capital structure adjustment speed.

	(1)	(2)	(3)	(4)	(5)	(6)	(7)	(8)	(9)
	*ΔLev*								
	Upward deviation in capital structure			Downward deviation in capital structure			Full sample		
*Dev*	0.412***	0.410***	0.419***	0.387***	0.386***	0.388***			
	(29.068)	(30.284)	(30.761)	(28.783)	(29.794)	(29.531)			
*Dev×Dum*	0.179***			-0.008					
	(13.361)			(-0.649)					
*Dev×Num*		0.128***			-0.003				
		(16.966)			(-0.529)				
*Dev×Ratio*			1.053***			-0.053			
			(14.994)			(-0.851)			
*Dev×D* ^*above*^							0.356***	0.370***	0.371***
							(24.117)	(27.211)	(27.366)
*D* ^*above*^							-0.032***	-0.030***	-0.032***
							(-13.941)	(-14.015)	(-15.102)
*Dum×Dev×D* ^*above*^							0.179***		
							(9.436)		
*Dum×D* ^*above*^							0.000		
							(0.058)		
*Num×Dev×D* ^*above*^								0.105***	
								(10.117)	
*Num×D* ^*above*^								-0.003	
								(-1.640)	
*Ratio×Dev×D* ^*above*^									0.953***
									(10.093)
*Ratio×D* ^*above*^									0.001
									(0.084)
*_cons*	0.045	0.043	0.040	0.036	0.036	0.036	0.067***	0.065***	0.064***
	(1.421)	(1.375)	(1.291)	(1.257)	(1.252)	(1.244)	(3.687)	(3.589)	(3.514)
Year	YES	YES	YES	YES	YES	YES	YES	YES	YES
Firm	YES	YES	YES	YES	YES	YES	YES	YES	YES
Industry	YES	YES	YES	YES	YES	YES	YES	YES	YES
Observations	12285	12285	12285	13716	13716	13716	26001	26001	26001
*R* ^*2*^	0.170	0.179	0.174	0.096	0.096	0.096	0.161	0.164	0.161

Note(s): *p < 0.1

**p < 0.05

***p < 0.01.

#### 4.3.4 The effect of ownership structure on the relationship between NCLSs and the dynamic capital structure adjustment

Table 8 reports the regression results on the effect of ownership structure on the relationship between NCLSs and dynamic capital structure adjustment. The results in columns (1)-(6) of Table 8 show that the coefficients of *Dev* are always significantly positive at the 1% level for both SOEs and NSOEs, and the coefficients of *Dev×Dum*, *Dev×Num*, and *Dev×Ratio* are also significantly positive at the 1% level, indicating that NCLSs of both SOEs and NSOEs significantly increase the dynamic capital structure adjustment speed. However, the results in columns (7)-(9) of Table 8 show that the coefficients of *Dev×Dum×NSoe*, *Dev×Num×NSoe*, and *Dev×Ratio×NSoe* are 0.057, 0.022, and 0.324, respectively, and are significant at the 1% level, indicating that compared with SOEs, NCLSs in NSOEs have a more significant positive impact on the dynamic capital structure adjustment speed, i.e., H4 is verified.

**Table 8:** The effect of ownership structure on the relationship between NCLSs and dynamic capital structure adjustment.

	(1)	(2)	(3)	(4)	(5)	(6)	(7)	(8)	(9)
	*ΔLev*								
	SOEs			NSOEs			Full sample		
*Dev*	0.298***	0.296***	0.308***	0.422***	0.422***	0.425***	0.360***	0.363***	0.370***
	(29.673)	(30.779)	(31.486)	(40.832)	(44.291)	(44.606)	(49.230)	(53.233)	(53.908)
*Dev×Dum*	0.092***			0.063***			0.039***		
	(7.815)			(5.334)			(3.129)		
*Dev×Num*		0.064***			0.041***			0.031***	
		(9.614)			(6.624)			(4.362)	
*Dev×Ratio*			0.393***			0.351***			0.130**
			(6.838)			(5.908)			(2.140)
*Dev×Dum×NSOE*							0.057***		
							(4.173)		
*Dev×Num×NSOE*								0.022***	
								(2.756)	
*Dev×Ratio×NSOE*									0.324***
									(4.611)
*_cons*	0.017	0.013	0.012	0.013	0.016	0.015	0.015	0.016	0.014
	(0.787)	(0.632)	(0.548)	(0.458)	(0.559)	(0.521)	(0.816)	(0.892)	(0.799)
Year	YES	YES	YES	YES	YES	YES	YES	YES	YES
Firm	YES	YES	YES	YES	YES	YES	YES	YES	YES
Industry	YES	YES	YES	YES	YES	YES	YES	YES	YES
Observations	10126	10126	10126	15875	15875	15875	26001	26001	26001
Adjusted *R*^*2*^	0.056	0.059	0.054	0.065	0.066	0.065	0.063	0.064	0.062

Note(s): *p < 0.1; **p < 0.05; ***p < 0.01.

### 4.4 Endogeneity test

#### 4.4.1 Propensity score matching (PSM) approach

**Fig 2:**
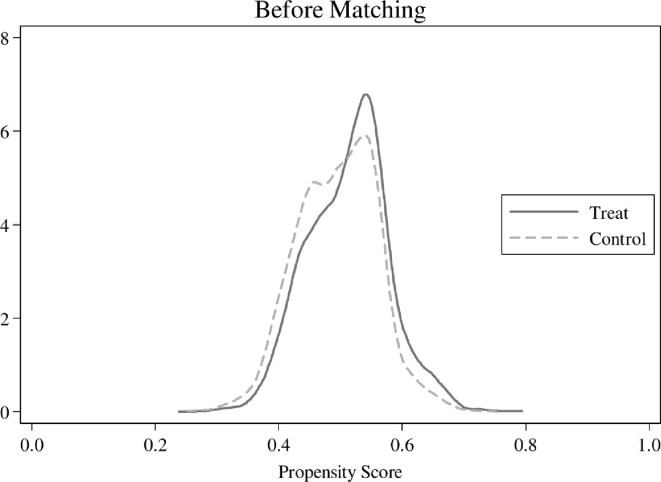
Density before pairing.

**Fig 3:**
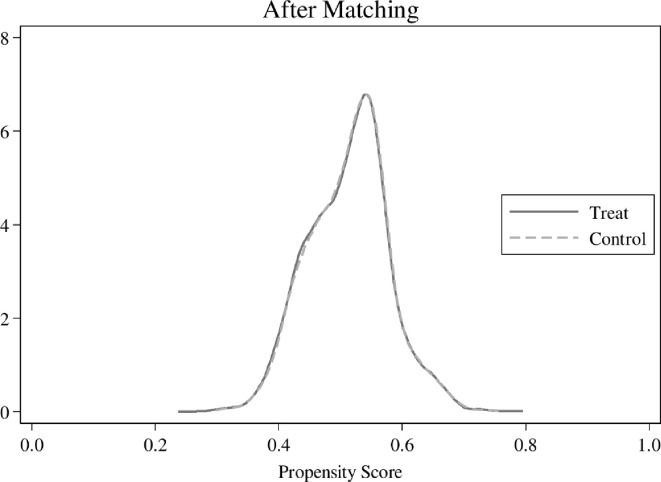
Density after pairing.

In the previous empirical tests, this paper uses a panel fixed effects model to address the endogeneity problem caused by unobservable firm characteristics. We utilize the PSM approach to further mitigate the problem of selection bias caused by observable firm characteristics. Based on the presence or absence of NCLSs, this paper divides the full sample into treatment group (*Dum* = 1) and control group (*Dum* = 0) and performs 1:1 nearest neighbor matching with the control variable in model (2) as the matching variable, and obtains 20158 observations after matching. As shown in Fig 2 Density before pairing and Fig 3 Density after pairing, there are some characteristic differences between the treatment and control groups before matching, while the density graphs of the two almost overlap after matching. The results of the balance test reported in Table 9 show that the absolute values of the standardized bias for the PSM sample are less than 2.3%, and in contrast to the original sample, there is little difference in the mean values of most of the variables between the two groups in the PSM model. We re-estimate model (4) using the PSM sample, and the results are presented in Table 10. We find that the regression coefficients of *Dev* are always significantly positive at the 1% level, and the regression coefficients of *Dev×Dum*, *Dev×Num*, and *Dev×Ratio* are all significantly positive at the 1% level, consistent with the previous results.

**Table 9:** Comparison of matching variables.

	(1)	(2)	(3)	(4)	(5)		(6)	(7)	(8)	(3)
	The original sample					The PSM sample				Reduction in bias (%)
	Treatment group	Control group	Diff. in means (t-stat)	Standardized bias (%)	Treatment group		Control group	Diff. in means (t-stat)	Standardized bias (%)	
*Size*	22.185	22.085	0.100***	7.8	22.185		22.163	0.022	1.7	78.6
*Roa*	0.040	0.040	0.000	0.9	0.040		0.040	0.000	1.4	-60.1
*Growth*	0.205	0.175	0.030***	6.8	0.205		0.215	-0.010*	-2.3	66.3
*Dep*	0.362	0.379	-0.017***	-9.6	0.362		0.363	-0.001	-0.6	93.6
*Pota*	0.020	0.020	-0.000	-0.9	0.020		0.020	-0.000	-0.3	67.8
*Indep*	0.415	0.420	-0.005***	-4.9	0.415		0.416	-0.001	-1.2	74.6

Note(s): *p < 0.1

**p < 0.05

***p < 0.01.

**Table 10:** Propensity score matching (PSM) approach.

	(1)	(2)	(3) (4)
	*ΔLev*		
*Dev*	0.358***	0.368***	0.382***
	(36.662)	(42.932)	(44.061)
*Dev×Dum*	0.093***		
	(8.944)		
*Dev×Num*		0.051***	
		(10.041)	
*Dev×Ratio*			0.345***
			(7.271)
*_cons*	0.010	0.011	0.009
	(0.449)	(0.493)	(0.408)
Year	YES	YES	YES
Firm	YES	YES	YES
Industry	YES	YES	YES
Observations	20158	20158	20158
Adjusted *R*^*2*^	0.042	0.043	0.040

Note(s): *p < 0.1

**p < 0.05

***p < 0.01.

#### 4.4.2 Two-stage least squares (2SLS) regression

We conduct the 2SLS regression to tackle endogeneity due to reverse causality and omitted variables. Referring to the idea of constructing instrumental variables in the existing literature [60,61], we select the following two instrumental variables: (a) the percentage of firms with NCLSs in the industry to which the firm belongs (*IV1*), (b) the percentage of firms with NCLSs in the province where the firm is located (*IV2*). Table 11 shows the results of 2SLS regression, from the first stage estimation results in column (1), column (3), and column (5), both the regression coefficients of *Dev×IV1* and *Dev×IV2* are significantly positive at the 1% level. The results of the validity tests of the instrumental variables are as follows: (a) The F-statistics of the first stage are 246.63, 202.06, and 214.03. (b) The Anderson canon. corr. LM statistics are 483.358, 397.554, and 420.666, rejecting the null hypothesis of the underidentification test. (c) The Cragg-Donald Wald F statistics are 246.631, 202.061, 214.032, rejecting the null hypothesis of the weak identification test. (d) The P-values of the Sargan statistics are 0.1482, 0.8759, and 0.1914, indicating that both *IV1* and *IV2* are exogenous. From the second-stage estimation results in column (2), column (4), and column (6) of Table 11, we find that the regression coefficients of *Dev* are always significantly positive at the 1% level, and the regression coefficients of *Dev×Dum*, *Dev×Num*, and *Dev×Ratio* are 0.387, 0.232, and 2.068, respectively, and all are significant at the 1% level, consistent with previous results.

**Table 11:** Two-stage least squares (2SLS) regression.

	(1)	(2)	(3)	(4)	(5)	(6)
	*Dev×Dum*	*ΔLev*	*Dev×Num*	*ΔLev*	*Dev×Ratio*	*ΔLev*
	first-stage	second-stage	first-stage	second-stage	first-stage	second-stage
*Dev×IV1*	1.142***		3.970***		0.584***	
	(4.851)		(8.952)		(12.291)	
*Dev×IV2*	0.835***		1.172***		0.104***	
	(18.888)		(14.074)		(11.698)	
*Dev*	-0.008	0.193***	-0.143***	0.208***	-0.016***	0.214***
	(-0.308)	(5.942)	(-2.891)	(6.978)	(-3.102)	(7.253)
*Dev×Dum*		0.387***				
		(6.532)				
*Dev×Num*				0.232***		
				(6.640)		
*Dev×Ratio*						2.068***
						(6.508)
Year	YES	YES	YES	YES	YES	YES
Firm	YES	YES	YES	YES	YES	YES
Industry	YES	YES	YES	YES	YES	YES
Observations	25895		25895		25895	
Centered *R*^*2*^	0.4055	0.1421	0.3169	0.1297	0.3245	0.1286
F-statistics	246.63		202.06		214.03	
Anderson canon. corr. LM statistic	483.358***		397.554***		420.666***	
Cragg-Donald Wald F statistic	246.631		202.061		214.032	
Sargan statistic	2.090		0.024		1.707	
Chi-sq(1) P-val	0.1482		0.8759		0.1914	

Note(s): *p < 0.1

**p < 0.05

***p < 0.01.

#### 4.4.3 Heckman two-stage model

Referring to Ben-Nasr et al. [32], we employ the Heckman two-stage selection model to address sample bias. In the first stage of the analysis, we use the two instrumental variables mentioned above, *IV1* and *IV2*. The first-stage regression results, presented in column (1) of Table 12, show that the regression coefficients of *IV1* and *IV2* are significantly positive at the 1% level. The second-stage regression results, presented in columns (2)-(4) of Table 12, show that the coefficients of the inverse Mills ratio (*Lambda*) are significantly negative at the 1% level. Additionally, the regression coefficients of *Dev* are always significantly positive at the 1% level, and the regression coefficients of *Dev×Dum*, *Dev×Num*, and *Dev×Ratio* are 0.079, 0.047, and 0.340, respectively, and all are significant at the 1% level, consistent with previous results.

**Table 12:** Heckman two-stage selection model.

	(1)	(2)	(3)	(4)
	*Dum*	*ΔLev*		
	First stage	Second stage		
*IV1*	12.494***			
	(6.561)			
*IV2*	4.821***			
	(16.362)			
*Size*	0.034			
	(1.578)			
*Roa*	0.196			
	(0.681)			
*Growth*	0.069***			
	(2.645)			
*Dep*	-0.431***			
	(-3.290)			
*Pota*	-0.484			
	(-0.276)			
*Indlev*	-0.593			
	(-1.230)			
*Dev*		0.361***	0.364***	0.372***
		(49.505)	(53.386)	(54.299)
*Dev×Dum*		0.079***		
		(9.303)		
*Dev×Num*			0.047***	
			(10.556)	
*Dev×Ratio*				0.340***
				(8.129)
*Lambda*		-0.008***	-0.008***	-0.008***
		(-8.511)	(-8.607)	(-8.380)
*_cons*	-3.599***	0.018	0.018	0.016
	(-6.102)	(0.992)	(0.994)	(0.904)
Year	YES	YES	YES	YES
Firm	YES	YES	YES	YES
Industry	YES	YES	YES	YES
Observations	26001	26001	26001	26001
Wald chi^2^/Adjusted *R*^*2*^	599.95	0.065	0.066	0.064

Note(s): *p < 0.1

**p < 0.05

***p < 0.01.

### 4.5 Robustness test

#### 4.5.1 Alternative measures of capital structure

In the previous section, the ratio of total liabilities to total assets is adopted as a measure of capital structure. Drawing on existing literature [15,62] and taking into account the actual situation of Chinese firms in adjusting their capital structure, we reconstruct the following two variables to measure capital structure: (a) the sum of short-term and long-term borrowings divided by total assets, (b) the interest-bearing debt divided by total assets. We recalculate the actual capital structure adjustment *ΔLev1* and *ΔLev2*, respectively. Then we recalculate the target capital structure and obtain the deviation of the capital structure from the target *Dev1* and *Dev2*, respectively. The regression results presented in Table 13 show that both the regression coefficients of *Dev1* and *Dev2* are always significantly positive at the 1% level, and the regression coefficients of *Dev1*×*Dum*, *Dev1*×*Num*, and *Dev1*×*Ratio*, *Dev2*×*Dum*, *Dev2*×*Num*, and *Dev2*×*Ratio* are all significantly positive at the 1% level, consistent with previous results.

**Table 13:** Alternative measures of capital structure.

	(1)	(2)	(3)	(4)	(5)	(6)
	*ΔLev1*			*ΔLev2*		
*Dev1*	0.450***	0.449***	0.460***			
	(61.579)	(65.613)	(67.171)			
*Dev1×Dum*	0.040***					
	(4.586)					
*Dev1×Num*		0.028***				
		(5.749)				
*Dev1×Ratio*			0.125***			
			(2.910)			
*Dev2*				0.387***	0.387***	0.394***
				(54.613)	(58.128)	(59.212)
*Dev2×Dum*				0.040***		
				(4.748)		
*Dev2×Num*					0.026***	
					(5.652)	
*Dev2×Ratio*						0.154***
						(3.752)
*_cons*	0.016	0.016	0.015	0.021	0.021	0.020
	(1.113)	(1.111)	(1.049)	(1.399)	(1.387)	(1.339)
Year	YES	YES	YES	YES	YES	YES
Firm	YES	YES	YES	YES	YES	YES
Industry	YES	YES	YES	YES	YES	YES
Observations	26001	26001	26001	26001	26001	26001
Adjusted *R*^*2*^	0.113	0.114	0.113	0.070	0.071	0.070

Note(s): *p < 0.1

**p < 0.05

***p < 0.01.

#### 4.5.2 Modified adjustment model

Model (1) includes both active adjustment and mechanical adjustment. Given that active adjustments are required to be explored by the dynamic trade-off theory, we distinguish between mechanical and active capital structure adjustments, Referring to Faulkender et al. [17], model (1) is modified to obtain model (8):

$$Lev_{i,t}-Le{v^{p}}_{i,t‐1}=\theta (Le{v^{*}}_{i,t}-Le{v^{p}}_{i,t‐1})+\varepsilon_{i,t}$$ (8)

$$Le{v^{p}}_{i,t‐1}=Debt_{i,t‐1}/(Asset_{i,t‐1}-NP_{i,t‐1})$$ (9)

where *Debt*_*i*,*t-1*_, *Asset*_*i*,*t-1*_, and *NP*_*i*,*t-1*_ represent the total liabilities, total assets, and net income of firm i in year t-1, respectively. If the firm does not have an active adjustment, then the capital structure of firm i in year t will be *Lev*^*p*^_*i*,*t-1*_, *and Lev*_*i*,*t*_ *-Lev*^*p*^_*i*,*t-1*_ will represent the active adjustment portion. To examine the effect of the NCLSs on the firm’s active adjustment, model (4) is modified to obtain model (10):

$$Lev_{i,t}-Le{v^{p}}_{i,t‐1}=(\kappa_{0}+\kappa_{1}\times Ncls_{i,t})\times Dev2_{i,t}+\varepsilon_{i,t}$$ (10)

where *Dev3* = Lev^*^_i,*t*_−*Lev*^*p*^_*i*,*t-1*_, denotes the difference obtained from the firm’s target capital structure after excluding the mechanical adjustment component. If the NCLSs can significantly increase the speed of the firm’s active adjustment, then the coefficient *κ*_1_ of *Ncls*_*i*,*t*_×*Dev3*_*i*,*t*_ in model (10) should be significantly positive. The regression results presented in Table 14 show that the regression coefficients of *Dev3* are always significantly positive at the 1% level, indicating that the sample firms do have an active adjustment toward the target capital structure, and the regression coefficients of *Dev3*×*Dum*, *Dev3*×*Num*, and *Dev3*×*Ratio* are all significantly positive at the 1% level, indicating that the NCLSs increase the firms’ active capital structure adjustment speed, consistent with previous results.

**Table 14:** The effect of NCLSs on the active capital structure adjustment speed.

	(1)	(2)	(3)
	*Lev*_*i*,*t*_ *-Lev*^*p*^_*i*,*t-1*_		
*Dev3*	0.401***	0.406***	0.418***
	(54.381)	(59.056)	(60.466)
*Dev3×Dum*	0.085***		
	(9.986)		
*Dev3×Num*		0.049***	
		(10.806)	
*Dev3×Ratio*			0.329***
			(7.811)
*_cons*	0.030	0.030	0.028
	(1.635)	(1.615)	(1.530)
Year	YES	YES	YES
Firm	YES	YES	YES
Industry	YES	YES	YES
Observations	26001	26001	26001
Adjusted *R*^*2*^	0.106	0.107	0.104

Note(s): *p < 0.1

**p < 0.05

***p < 0.01.

#### 4.5.3 Controlling for variables related to corporate governance

Studies have shown that the level of corporate governance is also a factor influencing the capital structure of firms and its optimization [63]. Therefore, this paper controls for corporate governance indicators such as executive compensation (*Mpay*: the natural logarithm of the total compensation of the top three executives), controlling shareholders’ shareholding ratio (*First*: the sum of the shareholding ratios of controlling shareholders and their concert parties), the proportion of independent directors (*Indep*: the ratio of the number of independent directors to the number of directors), and ownership structure (*Soe*: SOEs take the value of 1, otherwise 0) and then recalculates target capital structure. We obtain the deviation of the capital structure from the target (*Dev4*). The results presented in Table 15 show that the regression coefficient of *Dev4* is always significantly positive at the 1% level, and the regression coefficients of *Dev4×Dum*, *Dev4×Num*, and *Dev4×Ratio* are all significantly positive at the 1% level, indicating that the conclusions of this paper still hold after controlling for the effects of corporate governance level variables on capital structure adjustment.

**Table 15:** The effect of NCLSs on the capital structure adjustment speed after controlling for corporate governance variables.

	(1)	(2)	(3)
	*ΔLev*		
*Dev4*	0.376***	0.375***	0.383***
	(50.170)	(53.528)	(54.323)
*Dev4×Dum*	0.066***		
	(7.672)		
*Dev4×Num*		0.045***	
		(9.766)	
*Dev4×Ratio*			0.321***
			(7.435)
*_cons*	0.015	0.016	0.014
	(0.848)	(0.878)	(0.789)
Year	YES	YES	YES
Firm	YES	YES	YES
Industry	YES	YES	YES
Observations	25505	25505	25505
Adjusted *R*^*2*^	0.062	0.063	0.062

Note(s): *p < 0.1

**p < 0.05

***p < 0.01.

#### 4.5.4 Excluding the impact of deleveraging policy

Since December 2015, the Central Committee of the Communist Party of China and the ministries and commissions have issued several policy documents on deleveraging intensively. The deleveraging policy is aimed at strictly controlling the excessive growth of debt scale in China to achieve long-term sustainable economic growth. Firms, as the most important participants of market economic activities, are naturally the core areas for the implementation of deleveraging policy. Therefore, the implementation of mandatory deleveraging in China is bound to have an impact on corporate capital structure decisions. To exclude the effect of the deleveraging policy, this paper tests the sample before the implementation of the deleveraging policy (from 2010 to 2015). The regression results presented in Table 16 show that the regression coefficient of *Dev* is always significantly positive at the 1% level, and the regression coefficients of *Dev*×*Dum*, *Dev*×*Num*, and *Dev*×*Ratio* are all significantly positive at the 1% level, and the results remain consistent with the previous paper.

**Table 16:** Excluding the impact of deleveraging policy.

	(1)	(2)	(3)
	*ΔLev*		
*Dev*	0.584***	0.598***	0.591***
	(52.304)	(57.943)	(56.921)
*Dev×Dum*	0.063***		
	(5.064)		
*Dev×Num*		0.025***	
		(4.017)	
*Dev×Ratio*			0.309***
			(5.050)
*_cons*	-0.055**	-0.055**	-0.055**
	(-1.997)	(-2.006)	(-1.987)
Year	YES	YES	YES
Firm	YES	YES	YES
Industry	YES	YES	YES
Observations	16756	16756	16756
Adjusted *R*^*2*^	0.113	0.112	0.113

Note(s): *p < 0.1

**p < 0.05

***p < 0.01.

### 4.6 Mechanism test

The above theoretical analysis suggests that NCLSs have the motivation and capability to actively engage in corporate governance, thus alleviating the problem of capital structure deviation and slow adjustment caused by agency costs or financing constraints. To verify the above two mechanisms, we examine the impact of NCLSs on agency costs and financing constraints, respectively. We set a dummy variable *Cost* to measure the agency cost, if the firm’s free cash flow is higher than the industry median for the year and the sales growth rate is lower than the industry median, the agency cost is the highest [64], and Cost takes the value of 1, and 0 otherwise. Referring to Kaplan and Zingales [65], we calculate the KZ index to measure the degree of financing constraints, and a larger *KZ* indicates a more severe degree of financing constraints.

The results of the mechanism test are reported in Table 17. Columns (1)-(3) of Table 17 Panel A show that the regression coefficients of *Dum*, *Num*, and *Ratio* are all significantly negative with *Cost* as the explanatory variable, indicating that NCLSs are effective in reducing agency costs. Columns (4)-(6) of Table 17 Panel A show that the regression coefficients of *Dum*, *Num*, and *Ratio* are all significantly negative with *KZ* as the explanatory variable, indicating that NCLSs are effective in inhibiting the financing constraints. The results of Table 17 Panel B show that the regression coefficients of *Dev×Dum*, *Dev×Num*, and *Dev×Ratio* are all significantly positive, and the above results verify that reducing agency costs and mitigating financing constraints serve as the important channels through which NCLSs influence the dynamic adjustment of capital structure.

**Table 17:** Mechanism test.

Panel A: Step 2 of the mediated effects model						
	(1)	(2)	(3)	(4)	(5)	(6)
	*Cost*			*KZ*		
	Channel test for agency costs			Channel test for financing constraints		
*Dum*	-0.263***			-0.112***		
	(-5.528)			(-6.855)		
*Num*		-0.217***			-0.109***	
		(-7.746)			(-11.755)	
*Ratio*			-2.047***			-1.043***
			(-7.726)			(-11.635)
*Size*	0.605***	0.613***	0.611***	0.496***	0.495***	0.495***
	(13.245)	(13.394)	(13.356)	(32.727)	(32.772)	(32.746)
*Roa*	3.256***	3.301***	3.273***	-5.394***	-5.384***	-5.387***
	(7.526)	(7.615)	(7.558)	(-34.120)	(-34.139)	(-34.154)
*Growth*	-0.162***	-0.154***	-0.154***	0.017	0.021	0.021
	(-4.153)	(-3.953)	(-3.939)	(1.202)	(1.498)	(1.506)
*Dep*	-0.785***	-0.810***	-0.801***	0.909***	0.896***	0.902***
	(-3.945)	(-4.067)	(-4.022)	(12.945)	(12.779)	(12.867)
*Pota*	23.833***	23.816***	23.587***	-4.579***	-4.697***	-4.795***
	(8.496)	(8.485)	(8.393)	(-4.619)	(-4.749)	(-4.846)
*Indlev*	-0.719	-0.744	-0.731	0.877***	0.876***	0.869***
	(-1.105)	(-1.142)	(-1.122)	(3.894)	(3.895)	(3.865)
*_cons*				-10.122***	-10.085***	-10.061***
				(-24.663)	(-24.632)	(-24.567)
Year	YES	YES	YES	YES	YES	YES
Firm	YES	YES	YES	YES	YES	YES
Industry	YES	YES	YES	YES	YES	YES
Observations	22160	22160	22160	23594	23594	23594
LR chi^2^ / *R*^*2*^	377.43	408.02	407.63	0.144	0.148	0.148
Panel B: Step 3 of the mediated effects model						
	(1)	(2)	(3)	(4)	(5)	(6)
	*ΔLev*					
	Channel test for agency costs			Channel test for financing constraints		
*Dev*	0.386***	0.388***	0.397***	0.423***	0.416***	0.427***
	(51.264)	(54.815)	(55.875)	(49.610)	(51.206)	(52.258)
*Dev×Dum*	0.071***			0.052***		
	(8.403)			(5.816)		
*Dev×Num*		0.044***			0.041***	
		(9.735)			(8.606)	
*Dev×Ratio*			0.301***			0.264***
			(7.135)			(5.909)
*Dev×Cost*	-0.099***	-0.098***	-0.100***			
	(-13.423)	(-13.276)	(-13.424)			
*Dev×KZ*				-0.018***	-0.016***	-0.017***
				(-5.307)	(-4.782)	(-5.050)
*_cons*	0.016	0.016	0.014	0.019	0.019	0.018
	(0.874)	(0.881)	(0.793)	(0.863)	(0.866)	(0.838)
Year	YES	YES	YES	YES	YES	YES
Firm	YES	YES	YES	YES	YES	YES
Industry	YES	YES	YES	YES	YES	YES
Observations	25985	25985	25985	23594	23594	23594
Adjusted *R*^*2*^	0.068	0.069	0.067	0.072	0.074	0.072

Note(s): *p < 0.1

**p < 0.05

***p < 0.01.

## 5. Conclusions and recommendations

This paper empirically examines the impact of NCLSs on corporate capital structure adjustment, using a sample of A-share listed firms in China from 2010 to 2020, and finds that NCLSs significantly increase the dynamic capital structure adjustment speed and reduce the deviation of the actual capital structure from the target capital structure. After distinguishing the different directions of deviation from the actual capital structure and the adjustment methods, we find that the governance effect of NCLSs on dynamic capital structure adjustment speed is asymmetric, showing that NCLSs significantly increase the speed of downward adjustment after upward deviation from the capital structure compared with the speed of upward adjustment after downward deviation from the capital structure. Based on the special economic system background in China, this paper further introduces the ownership structure as the moderating variable and finds that the NCLSs significantly increase the dynamic capital structure adjustment speed in both SOEs and NSOEs, but the positive effect of NCLSs on the dynamic capital structure adjustment speed is more significant in NSOEs than in SOEs. The mechanism analysis suggests that reducing agency costs and mitigating financing constraints are important mechanisms through which NCLSs influence the dynamic adjustment of capital structure.

Based on the above findings, this paper puts forward the following policy recommendations: First, listed firms should pay attention to the governance role of NCLSs when designing or reforming their ownership structure, increase the introduction of NCLSs, especially for listed firms controlled by a single large shareholder, and actively introduce NCLSs to further optimize the firm’s ownership structure, while giving NCLSs more opportunities to gain a deeper understanding of the listed firm and be actively guided to participate in corporate governance. Second, government departments should continue to actively and steadily promote mixed ownership reform, encourage the expansion of institutional investors, and comprehensively deepen the high-level opening of the capital market to the outside world, to attract more high-quality external investors to participate in corporate governance. The regulatory authorities should further strengthen daily supervision to regulate the content and procedures of information disclosure of listed firms enhance the understanding of NCLSs to listed firms, and further improve the institutional mechanism of investor protection to create favorable conditions to effectively protect the legitimate rights and interests of NCLSs and increase their willingness to participate in corporate governance. Third, NCLSs should actively participate in corporate governance, express their demands, and protect their legitimate rights and interests. In addition, NCLSs need to learn and absorb new professional knowledge and skills, so that they can accurately judge whether the business strategy decisions made by controlling shareholders and managers are scientifically appropriate and avoid blind interference, which provides new ideas to solve problems for the firm and reduce the risk of decision-making errors.

## Supporting information

S1 Data (ZIP)
