# Supplementary material for: Non-controlling large shareholders and dynamic capital structure adjustment in China
Source: PLoS One. 2024 Jul 31;19(7):e0307066. doi: 10.1371/journal.pone.0307066 (PMC11290624; doi:10.1371/journal.pone.0307066)
Supplement: S1 Data — (ZIP) [file pone.0307066.s001.zip › Data/result/BL.rtf]

	(1)	
	Xdlev4zWBBL5	
Xdlev4zIV1	0.584***	
	(12.291)	
		
Xdlev4zIV2	0.104***	
	(11.698)	
		
Xdlev4z	-0.016***	
	(-3.102)	
		
YEAR1	0.000	
	(0.914)	
		
YEAR2	0.001	
	(1.580)	
		
YEAR3	0.001***	
	(2.665)	
		
YEAR4	0.000	
	(1.137)	
		
YEAR5	0.001	
	(1.514)	
		
YEAR6	-0.000	
	(-0.059)	
		
YEAR7	-0.000	
	(-0.506)	
		
YEAR8	0.000	
	(0.556)	
		
YEAR9	0.000	
	(0.661)	
		
YEAR10	0.000	
	(0.606)	
		
YEAR11	0.000	
	(.)	
		
INDS1	-0.007**	
	(-2.146)	
		
INDS2	-0.003	
	(-1.540)	
		
INDS3	0.000	
	(0.030)	
		
INDS4	-0.000	
	(-0.025)	
		
INDS5	-0.000	
	(-0.171)	
		
INDS6	-0.002	
	(-1.021)	
		
INDS7	0.002	
	(1.011)	
		
INDS8	-0.002	
	(-0.949)	
		
INDS9	0.001	
	(0.470)	
		
INDS10	0.005**	
	(2.363)	
		
INDS11	0.006	
	(1.354)	
		
INDS12	-0.001	
	(-0.476)	
		
INDS13	0.001	
	(0.800)	
		
INDS14	0.003	
	(1.609)	
		
INDS15	-0.005*	
	(-1.701)	
		
INDS16	-0.001	
	(-0.723)	
		
INDS17	0.000	
	(0.084)	
		
INDS18	-0.015***	
	(-3.966)	
		
INDS19	-0.000	
	(-0.041)	
		
INDS20	-0.001	
	(-0.268)	
		
INDS21	0.000	
	(.)	
N	25895	
r2		
r2_a		
F		
t statistics in parentheses
* p < 0.1, ** p < 0.05, *** p < 0.01
