# Supplementary material for: Non-controlling large shareholders and dynamic capital structure adjustment in China
Source: PLoS One. 2024 Jul 31;19(7):e0307066. doi: 10.1371/journal.pone.0307066 (PMC11290624; doi:10.1371/journal.pone.0307066)
Supplement: S1 Data — (ZIP) [file pone.0307066.s001.zip › Data/result/Dum.rtf]

	(1)	
	Xdlev4zWBDum5	
Xdlev4zIV1	1.142***	
	(4.851)	
		
Xdlev4zIV2	0.835***	
	(18.888)	
		
Xdlev4z	-0.008	
	(-0.308)	
		
YEAR1	0.003	
	(1.611)	
		
YEAR2	0.004*	
	(1.885)	
		
YEAR3	0.006***	
	(3.300)	
		
YEAR4	0.003	
	(1.494)	
		
YEAR5	0.002	
	(0.952)	
		
YEAR6	0.000	
	(0.212)	
		
YEAR7	0.000	
	(0.111)	
		
YEAR8	0.001	
	(0.887)	
		
YEAR9	0.002	
	(1.083)	
		
YEAR10	0.002	
	(1.257)	
		
YEAR11	0.000	
	(.)	
		
INDS1	-0.046***	
	(-3.030)	
		
INDS2	-0.014	
	(-1.431)	
		
INDS3	-0.008	
	(-0.995)	
		
INDS4	-0.001	
	(-0.192)	
		
INDS5	-0.002	
	(-0.325)	
		
INDS6	-0.008	
	(-0.800)	
		
INDS7	0.008	
	(0.817)	
		
INDS8	-0.008	
	(-0.876)	
		
INDS9	0.001	
	(0.203)	
		
INDS10	0.010	
	(0.918)	
		
INDS11	0.034	
	(1.514)	
		
INDS12	-0.007	
	(-0.951)	
		
INDS13	0.007	
	(1.007)	
		
INDS14	0.023***	
	(2.682)	
		
INDS15	-0.040***	
	(-2.915)	
		
INDS16	0.014	
	(1.484)	
		
INDS17	0.004	
	(0.225)	
		
INDS18	-0.037**	
	(-2.030)	
		
INDS19	-0.011	
	(-0.647)	
		
INDS20	-0.006	
	(-0.546)	
		
INDS21	0.000	
	(.)	
N	25895	
r2		
r2_a		
F		
t statistics in parentheses
* p < 0.1, ** p < 0.05, *** p < 0.01
