# Supplementary material for: Non-controlling large shareholders and dynamic capital structure adjustment in China
Source: PLoS One. 2024 Jul 31;19(7):e0307066. doi: 10.1371/journal.pone.0307066 (PMC11290624; doi:10.1371/journal.pone.0307066)
Supplement: S1 Data — (ZIP) [file pone.0307066.s001.zip › Data/result/Num.rtf]

	(1)	
	Xdlev4zWBNum5	
Xdlev4zIV1	3.970***	
	(8.952)	
		
Xdlev4zIV2	1.172***	
	(14.074)	
		
Xdlev4z	-0.143***	
	(-2.891)	
		
YEAR1	-0.002	
	(-0.479)	
		
YEAR2	0.002	
	(0.511)	
		
YEAR3	0.006*	
	(1.899)	
		
YEAR4	-0.000	
	(-0.099)	
		
YEAR5	-0.000	
	(-0.015)	
		
YEAR6	0.000	
	(0.140)	
		
YEAR7	-0.002	
	(-0.557)	
		
YEAR8	0.002	
	(0.529)	
		
YEAR9	0.002	
	(0.796)	
		
YEAR10	0.003	
	(0.982)	
		
YEAR11	0.000	
	(.)	
		
INDS1	-0.081***	
	(-2.794)	
		
INDS2	-0.026	
	(-1.418)	
		
INDS3	-0.017	
	(-1.091)	
		
INDS4	-0.015	
	(-1.147)	
		
INDS5	-0.010	
	(-0.761)	
		
INDS6	-0.027	
	(-1.478)	
		
INDS7	-0.000	
	(-0.016)	
		
INDS8	-0.009	
	(-0.520)	
		
INDS9	0.006	
	(0.470)	
		
INDS10	0.034*	
	(1.706)	
		
INDS11	0.011	
	(0.274)	
		
INDS12	-0.001	
	(-0.039)	
		
INDS13	0.007	
	(0.517)	
		
INDS14	0.026	
	(1.594)	
		
INDS15	-0.029	
	(-1.134)	
		
INDS16	-0.021	
	(-1.198)	
		
INDS17	-0.027	
	(-0.778)	
		
INDS18	-0.064*	
	(-1.862)	
		
INDS19	-0.003	
	(-0.084)	
		
INDS20	0.012	
	(0.604)	
		
INDS21	0.000	
	(.)	
N	25895	
r2		
r2_a		
F		
t statistics in parentheses
* p < 0.1, ** p < 0.05, *** p < 0.01
