# Supplementary material for: Non-controlling large shareholders and dynamic capital structure adjustment in China
Source: PLoS One. 2024 Jul 31;19(7):e0307066. doi: 10.1371/journal.pone.0307066 (PMC11290624; doi:10.1371/journal.pone.0307066)
Supplement: S1 Data — (ZIP) [file pone.0307066.s001.zip › Data/result/Table2.rtf]

Variable	N	Mean	p50	SD	Min	Max	
lev w	26000	0.441	0.437	0.203	0.0530	0.869	
Ydlev	26000	0.0100	0.00800	0.0820	-0.801	0.802	
Xdlev4z	26000	0.0100	0.0110	0.155	-0.665	0.512	
WBDum5	26000	0.504	1	0.500	0	1	
WBNum5	26000	0.736	1	0.902	0	9	
WBBL5	26000	0.0820	0.0500	0.106	0	0.808	
Size 1 w	26000	22.14	21.96	1.275	19.50	25.79	
RoA 1 w	26000	0.0400	0.0360	0.0510	-0.160	0.193	
Growth 1 w	26000	0.190	0.113	0.446	-0.567	2.905	
Pota 1 w	26000	0.371	0.357	0.181	0.0260	0.816	
Ndts1 1 w	26000	0.0200	0.0160	0.0150	0	0.0710	
lev 1ind w	26000	0.417	0.396	0.104	0.229	0.693	
