# Supplementary material for: Non-controlling large shareholders and dynamic capital structure adjustment in China
Source: PLoS One. 2024 Jul 31;19(7):e0307066. doi: 10.1371/journal.pone.0307066 (PMC11290624; doi:10.1371/journal.pone.0307066)
Supplement: S1 Data — (ZIP) [file pone.0307066.s001.zip › Data/result/Table3.docx]

Table 3

|  | lev_w | Ydlev | Xdlev4z | WBDum5 | WBNum5 | WBBL5 | Size_1_w | RoA_1_w | Growth_1_w | Pota_1_w | Ndts1_1_w | lev_1ind_w |
| --- | --- | --- | --- | --- | --- | --- | --- | --- | --- | --- | --- | --- |
| lev_w | 1 | 0.159*** | -0.801*** | -0.010* | -0.017*** | 0.001 | 0.488*** | -0.396*** | 0.026*** | 0.294*** | -0.034*** | 0.357*** |
| Ydlev | 0.184*** | 1 | 0.188*** | 0.001 | -0.001 | -0.002 | -0.088*** | 0.009 | 0.019*** | -0.080*** | -0.052*** | -0.052*** |
| Xdlev4z | -0.786*** | 0.232*** | 1 | 0.013** | 0.022*** | 0.019*** | -0.188*** | 0.294*** | -0.035*** | -0.180*** | -0.009 | -0.195*** |
| WBDum5 | -0.012** | -0.003 | 0.013** | 1 | 0.945*** | 0.924*** | 0.021*** | 0.017*** | 0.023*** | -0.042*** | 0.006 | -0.017*** |
| WBNum5 | -0.025*** | -0.013** | 0.028*** | 0.810*** | 1 | 0.951*** | 0.025*** | 0.015** | 0.029*** | -0.043*** | 0.003 | -0.014** |
| WBBL5 | 0.008 | -0.009 | 0.026*** | 0.766*** | 0.861*** | 1 | 0.049*** | 0.012* | 0.017*** | -0.022*** | 0.026*** | 0.006 |
| Size_1_w | 0.486*** | -0.073*** | -0.127*** | 0.039*** | 0.043*** | 0.109*** | 1 | -0.074*** | 0.046*** | 0.155*** | -0.014** | 0.244*** |
| RoA_1_w | -0.340*** | 0.026*** | 0.229*** | 0.005 | -0.001 | 0.008 | -0.025*** | 1 | 0.295*** | -0.204*** | -0.057*** | -0.161*** |
| Growth_1_w | 0.050*** | 0.002 | -0.036*** | 0.034*** | 0.049*** | 0.030*** | 0.046*** | 0.187*** | 1 | -0.068*** | -0.113*** | -0.039*** |
| Pota_1_w | 0.304*** | -0.087*** | -0.160*** | -0.048*** | -0.045*** | -0.009 | 0.177*** | -0.154*** | -0.057*** | 1 | 0.516*** | 0.174*** |
| Ndts1_1_w | -0.011* | -0.054*** | -0.027*** | -0.004 | -0.007 | 0.030*** | 0.027*** | -0.066*** | -0.102*** | 0.508*** | 1 | -0.106*** |
| lev_1ind_w | 0.398*** | -0.045*** | -0.204*** | -0.024*** | -0.018*** | 0.011* | 0.277*** | -0.121*** | 0.018*** | 0.214*** | -0.165*** | 1 |
